# The Family Function and Exercise Behavior of Chinese College Students: A Moderated Mediation Model of Exercise Value Cognition and Only-Child Status

**DOI:** 10.3389/fpsyg.2021.644742

**Published:** 2021-08-27

**Authors:** Ming Wu, Pei-Yao Wu, Jian Yang, Xin Li

**Affiliations:** ^1^School of Physical Education (Main Campus), Zhengzhou University, Zhengzhou, China; ^2^Changzhou University Huaide College, Jingjiang, China; ^3^College of Physical Education and Health, East China Normal University, Shanghai, China

**Keywords:** family function, exercise value cognition, exercise behavior, only child, moderated mediation model, Chinese college students

## Abstract

The purpose of this study is to investigate the mediating role of exercise value cognition between family function (FF) and exercise behavior and the moderating role of an only-child status. A questionnaire survey was conducted on 504 Chinese college students using the FF scale, the exercise value cognition scale, and the exercise behavior scale. The analysis yielded four main findings. (1) There are significant differences between an only-child and a non-only-child for negative exercise behavior and FF. The only-child group has a higher average FF score and a lower average negative exercise score. (2) Exercise behavior and four of its dimensions—exercise autonomy, attention control, exercise planning, and situational induction—are each significantly positively correlated with FF and exercise value cognition. (3) FF is a significantly positive predictor of exercise behavior, both directly and through exercise value cognition, which plays a partial mediating role. (4) Only-child status significantly moderates the mediating effect of exercise value cognition in the link between FF and exercise behavior. The intergroup differences mainly manifest in the influence of FF on exercise behavior and the influence of exercise value cognition on exercise behavior. In the only-child subsample, exercise value cognition plays a complete mediating role. The results of the current study demonstrated the important role that FF and exercise value cognition played in promoting the exercise behavior of college students. These findings have important implications for exercise behavior in adolescents by maintaining sound communication between family members and developing a healthy lifestyle or value cognition.

## Introduction

Interpretation of scholars and the intervention angle on individual exercise behavior have gradually shifted from focusing on a single factor or the individual level to considering the entire social ecosystem. Family, the basic unit of society and one of the mesosocial ecosystems affecting exercise behavior (Schmidt et al., 2016; Kilanowski, 2017), provides crucial environmental support for the health education and healthy development of family members, and it is a key area of youth exercise promotion research. In the 1980s, China adopted the relatively strict one-child policy (OCP), aiming to control population growth, restructure population development, and boost national development and economic growth. This policy made only-child families become a common phenomenon in China. Under the influence of traditional cultures, economic growth, and OCP, the status of family members has gradually become equal, the network interactivity between families and family relatives are more frequent, and family life more colorful; meanwhile, old-age care or other functions of the traditional family have weakened or disappeared. Meanwhile, the educational philosophy of Chinese parents has gradually shifted from “intellectual development” to “comprehensive development”—a change that provides an opportunity for promoting exercise by college students at the family level. Based on these developments and against the background of China's social culture, this research explores the role of family function (FF) in physical exercise promotion for only-child and non-only-child college students. To this end, it combines the social ecology theory of exercise behavior with the FF process-orientation theory, thereby providing theoretical and practical support for promoting the healthy development of college students.

## Theory and Hypotheses

### Family Function and Exercise Behavior

Scholars classified the theories of FF into two categories: outcome-orientation theory and process-orientation theory (Miller et al., 1985; Beavers and Hampson, 2000; Schwab and Gray-Ice, 2000; Skinner and Steinhauer, 2000; Shek, 2002; Fang et al., 2004). Family function outcome-orientation theory classifies families according to the effects of FF, whereas family function process-orientation theory, based on family systems theory, focuses on the environmental conditions supporting the physical and mental health and the development of family members. The process-orientation theory, represented by the McMaster family functioning model (Miller et al., 1985) and family engineering model (Skinner and Steinhauer, 2000), posits that FF is an attribute of family group structure and a kind of interpersonal interaction, including problem-solving, communication, family intimacy, and other aspects. Smooth family functioning is positively correlated with the physical and mental health of family members (Miller et al., 1985; Skinner and Steinhauer, 2000). In the field of exercise behavior, numerous studies have confirmed the role of parents and family environment in cultivating and shaping the exercise behavior of children. For example, Sicilia et al. (2020) pointed out that the exercise awareness of parents is vital for cultivating exercise awareness and behavior in youths. Essiet et al. (2017) found that living with active siblings could increase the engagement of individuals in physical exercise. Miller et al. (2019) suggested that personal/social environmental factors, including family engagement in and support for physical exercise, are positively related to the engagement of individuals in medium-to-high intensity physical exercise. A similar study found a relationship between the physical exercise of family members and an individual, together with a positive correlation between more active family members and the participation of an individual in physical exercise (Martín-Matillas et al., 2011). However, existing research focuses on the influence of certain factors within the family system on individual exercise behavior. From the perspective of FF process-orientation theory, the discussion of concrete or abstract factors can neither fully explain the influence of the family system on individual exercise behavior nor the role of FF in this process. Although some studies have reported on how FF can improve the spontaneous physical exercise habits of an individual and reduce the sedentary time of adolescents (Egawa, 2013; Loprinzi, 2015), the mechanism through which FF influences the exercise behavior of college students is yet to be explored. Given the role of FF in bolstering individual physical and mental health and its impact on health-related habits (such as eating and physical exercise), the following hypothesis is proposed:

*Hypothesis 1: Family function has a significant predictive effect on the exercise behavior of college students*.

### Exercise Value Cognition

Exercise value cognition is the comprehension of an individual on the health benefits of exercise. As the concept of equal and democratic education has taken root in the minds of Chinese people, the education concept in China has gradually shifted back from school and performance-oriented education to the core of “cultural foundation,” “independent development,” and “social participation.” One study found that cultural capital cultivated in the family environment, as an endogenous resource, develops various skills, knowledge, and habits in children through individual learning, relevant knowledge comprehension, and practice (Liu, 2019). Both parenting style and parental exercise attitude have been identified as positive predictors of the exercise motivation and exercise attitude of the children (Rausch et al., 2015; Yaffe, 2018). Family environment, including support and expectation, is significantly correlated with individual behaviors (Dewar et al., 2013; Kim et al., 2015; Knol et al., 2016; such as career preparation, exercise, and obesity prevention). Chinese researchers insist that, with recent changes in society, Chinese families have gradually deepened their understanding of the educational and cultural value of physical exercise; this higher cognition of physical exercise value at the family level is bound to influence the cognition of physical exercise value on children (Zhu and Sun, 2016). In the field of physical exercise, individual exercise value cognition has been found to positively impact exercise attitude, motivation, and behavior. For example, Ferguson et al. (1989) found that factors like exercise value cognition and exercise attitude have a significantly positive impact on individual exercise behavior. Similarly, Maruf et al. (2017) identified that improving exercise value cognition and enriching exercise knowledge could improve the attitude of an individual toward participating in exercise, in turn generating or increasing their exercise behavior. Experimental studies have also concluded that the publicity of exercise knowledge and the supply of exercise-related educational resources could effectively improve the exercise value cognition of an individual, thereby improving their level of exercise motivation and promoting their participation in health-promoting exercise activities (Xiong and Liu, 2013; Cadilhac et al., 2015). Drawing on the three-factor model of social cognition theory, FF, and exercise value cognition could be expected to positively affect individual exercise behavior. On the one hand, the exercise knowledge base and exercise attitude among family members likely affects the exercise value cognition and exercise behavior of young people. On the other hand, enhancing individual exercise value cognition is conducive to engagement in exercise behavior. Based on the above, the following research hypotheses are proposed:

*Hypothesis 2: Family function has a significant predictive effect on the exercise value cognition of college students*.

*Hypothesis 3: Exercise value cognition has a significant predictive effect on exercise behavior*.

*Hypothesis 4: Exercise value cognition plays a mediating role in the influence of family function on the exercise behavior of college students*.

### Moderating the Role of Only-Child Status

Will the differences in family structure, intimacy, and resource acquisition between only-children and non-only-children moderate the mediated relationship between FF and exercise behavior *via* exercise value cognition? No prior research has directly investigated whether only-child status affects this particular mechanism. However, similar studies provide potentially relevant insights. According to the quantity–quality model of Becker and Lewis (1973), having fewer children enables each child in the family to obtain more resources, thereby improving the quality of the population (Zhao and Zhou, 2018). In China, parents throw more resources behind an only–child, as compared with non-only-children. Only-children benefit from higher family intimacy in the family system, a better parent–child relationship, and a higher level of FF (Chi et al., 2020). Chinese researchers have suggested that the health literacy level of only-children (including health-related beliefs, knowledge, lifestyle, and basic skills) exceeds that of non-only-children (Li et al., 2018). In addition, based on data from the China Education Panel Survey (CEPS) 2014–2015, Hu and Yu (2019) found that the education level of parents and family economic conditions strongly affected the participation of middle school students in exercise, and that only-child status boosted these relationships. It could be inferred that in the family system, favorable FF positively affect exercise value cognition and exercise behavior of children. Moreover, these effects are likely higher for only-children due to the higher level of FF and better performance in health literacy and exercise behavior. Based on the above analysis, the following hypothesis is proposed:

*Hypothesis 5: Only-child status moderates the mediating effect of exercise value cognition on the relationship between family function and the exercise behavior of college students*.

In summary, the purpose of this study is to investigate the mediating role of exercise value cognition between FF and exercise behavior and the moderating role of only-child status, by analyzing the relationship between FF, exercise behavior, and exercise value cognition.

## Materials and Methods

### Participants and Procedure

The survey was conducted in Henan Province, China. Three universities, namely Zheng Zhou University, Henan University, and Henan University of Technology, covering multiple fields and disciplines, were selected. The participants were undergraduate students (from freshmen to seniors). The electronic questionnaire was distributed to participants *via* QQ groups. It took approximately 15 min to answer all the questions. Out of the 570 college students who received the e-questionnaire, 550 returned the survey (96.49% response rate). To ensure the accuracy of the data and the sound operation of the structural equation model, the retrieved questionnaires were strictly screened to remove any missing responses, straight-line responses (answering each question with the same answer, such as “111111111…” or “222222222…”), and pattern responses (following a certain artificial rule, such as “7,6,5,4,3,2,1,7,6,5,4,3,2,1…” or “5,5,5,4,4,4,3,3,3,2,2,2,1,1,1…”). Finally, 504 valid questionnaires were obtained (88.42% effective response rate). The final sample comprised 165 men (32.74%) and 339 women (67.26%); 220 freshmen (43.65%) and 141 sophomores (27.98%); 96 juniors (19.05%) and 47 seniors (9.33%). The average age was 19.39 ± 1.29 years. A small gift (such as tips or vouchers) was given to each participant as an appreciation for participating in the survey. According to local legislation and institutional requirements, there is no need for the study to undergo ethical review or obtain signed informed consent forms from participants. However, a process to ensure that all participants have informed consent to participate is still included in the survey. When subjects enter the e-questionnaire, all the information in a document appeared on the first page. The document stated that the investigation was anonymous, and that its results would only be used for scientific research and would not pose any risk to them in their daily life. It also informed them that participation in the research was entirely voluntary. Subjects could only proceed to the questionnaire after confirming they had read the document and agreed to participate in the study. The method of protecting the privacy of the subjects has been presented in the Supplementary Materials.

### Measures

#### Family Function Scale

Family functions are greatly influenced by culture. This study adapted the McMaster Family Assessment Device (Epstein et al., 1983) to fit the characteristics of the Chinese family system. The FF Scale contains seven items (see Supplementary Materials), such as “Family members can share interests and hobbies with each other” and “Family members like to spend their spare time together.” Subjects were asked to express their level of agreement regarding the statements based on a seven-point Likert scale (“strongly disagree” to “strongly agree”). The FF scale's *Cronbach's* α is 0.83. Following the recommendations of DeVellis (2003), the internal quality of this scale was satisfactory. The CFA model demonstrated a satisfactory fit [normed chi square (*x*^2^*/df*) = 3.027, comparative fit index (CFI) = 0.985, goodness of fit index (GFI) = 0.975, Tucker–Lewis index (TLI) = 0.977, root mean square error of approximation (RMSEA) = 0.063, standardized root mean square residual (SRMR) = 0.024]. Following the recommendations of Schumacker and Lomax (2016), this scale was suitable for the sample studied.

#### Exercise Value Cognition Scale

This study adapted the physical exercise value cognition and exercise behavior questionnaire—value cognition subscale (Yao, 2013). According to research needs and the results of confirmatory factor analysis (CFA), four items were finally retained, which are: “I think physical exercise is a good fitness and recreational activity;” “I think physical exercise is good for physical and mental health;” “Physical exercise enriches my life;” “Physical exercise is one of my hobbies.” Subjects were asked to express their level of agreement regarding the statements based on a seven-point Likert scale (“strongly disagree” to “strongly agree”). Item scores were totaled to calculate the exercise value cognition (EVC) score—the higher the score, the greater the exercise value cognition of the individual. Cronbach's α of the EVC scale is 0.90. Following the recommendations of DeVellis (2003), the internal quality of this scale was satisfactory. The CFA model demonstrated a satisfactory fit (*x*^2^*/df* = 0.063, CFI = 1.000, GFI = 1.000, TLI = 1.004, RMSEA = 0.000, SRMR = 0.001). Following the recommendations of Schumacker and Lomax (2016), this scale was suitable for the sample studied.

#### Exercise Behavior Scale

The exercise behavior (EB) scale of this study draws on theories of exercise behavior and the exercise attitude scale—subscale of behavioral control and behavioral habits (Mao, 2003). The EB scale, initially containing 30 items, was subject to a pilot study by inviting 247 college students to complete and comment on the questionnaire. The results of the exploratory factor analysis (EFA) show that the Kaiser–Meyer–Olkin measure of sampling adequacy value is 0.922, total variance explained is 62.42%. After eliminating seven items with factor loading <0.5, the final EM Scale comprised 23 items across five dimensions (see Supplementary Materials for specific results):

- Autonomous exercise (AE): Individuals are willing to exercise and develop exercise habits.
- Attention control (AC): individuals are able to devote to the process of exercise.
- Exercise planning (EP): Individuals can make and stick to exercise plans.
- Situation induction (SI): Individuals often actively participate in exercise due to exams or health problems.
- Negative exercise (NE): Individuals withdraw from exercise after being thwarted and frustrated.

The EB scale consists of 23 items endorsed on a seven-point Likert scale indicating how true each item is for the students (see Supplementary Materials). Item scores were totaled to calculate the EB score: the higher the score, the better the exercise behavior of the individual (including AE, AC, EP, SI), lower the score the worse the NE of the individual. Cronbach's α of the EB scale is 0.91. Following the recommendations of DeVellis (2003), the internal quality of this scale was satisfactory. The CFA model demonstrated a satisfactory fit (*x*^2^*/df* = 3.084, CFI = 0.932, GFI = 0.890, TLI = 0.921, RMSEA = 0.064, SRMR = 0.053). Following the recommendations of Schumacker and Lomax (2016), this scale was suitable for the sample studied.

### Data Analysis

SPSS 21.0 and AMOS 22.0 were used to analyze the data. The descriptive statistics and independent sample *t*-test were used to analyze the difference in FF, exercise value cognition, and exercise behavior between only-child and non-only-child. In the independent sample *t*-test, only-child status was set as the independent variable, while FF, exercise value cognition, and exercise behavior (both overall and its five dimensions) were set as dependent variables. In the preliminary analysis phase, the reliability and validity of the scales were determined using Cronbach's alpha and average variance extracted after CFA, which was conducted in AMOS 22.0. On the basis of the hypothesis, a structural equation model (SEM) was used to determine the moderated mediation association between FF, exercise value cognition, exercise behavior, and only-child status. The hypothesis was tested in two steps.

First, the mediation model without the hypothesized interaction was assessed in only-child group, non-only-child group, and full sample. To better analyze the mediating role of exercise value cognition for only-child and non-only-child college students, mediation model (M1) were constructed. Following the recommendations of Jackson (2003) and Schreiber et al. (2006), six indicators, namely: *x*^2^*/df*, TLI, CFI, GFI, RMSEA, and SRMR were selected to evaluate model fit. To further examine the mediating effect of exercise value cognition, bootstrapping was used to test the significance of the mediating effects in M1 (MacKinnon et al., 2002, 2004). After first using repeated random sampling to extract 2,000 bootstrap samples from the original data, 2,000 estimated values of the mediating effect were generated, thereby forming an approximate sampling distribution. The 95% confidence interval (CI) for the mediating effect was estimated by taking the 2.5th percentile and the 97.5th percentile. If the CI does not contain 0, this indicates that the mediating effect is significant.

Second, the moderated mediation model with latent interaction was tested by using multigroup analysis. To analyze which path of the mediation model the only-child status moderates, a multigroup analysis technique was adopted. Taking only-child and non-only-child as group variables, nested models were constructed on the basis of the mediation model to conduct the multigroup invariance test. First, the unconstrained M2 (freely estimating each path coefficient) was constructed. Then, M3 (measurement weights of the two groups are equal) was built based on M2. Finally, based on M3, M4 was constructed (structural weights of the two groups are also equal). Further details of each model are provided in the Supplementary Materials. The chi-square difference value was adopted for differential analysis of the nested models. *p* < 0.05 was considered statistically significant.

Having identified the unbalanced gender composition of the sample during data analysis, we sought to ensure the scientific rigor of the study by testing for gender difference in the variables and testing the moderating effect by multigroup comparison (see Supplementary Materials).

## Results

### Common Method Variance Test

Process control and Harman's single-factor test were, respectively, used to minimize and examine common method bias. The process control steps included, for example, explaining the anonymity and confidentiality of completed questionnaire responses, and assuring that the data would only be used in academic research. The results of Harman's single-factor test show that the factors with initial eigenvalues >1 are explained between 5.63 and 20.83% of the variance; the average explained variance was 10.73% and the standard deviation was 5.48%. The maximum explanatory power of any factor is not two standard deviations (10.96%) greater than the average explanatory power. Therefore, it can be concluded that the survey is unaffected by the common method bias.

### Independent Sample *t*-Test

The results show significant differences between only-child and non-only-child for negative exercise (*p* = 0.038 < 0.05) and FF (*p* = 0.002 < 0.01), but not for EB overall, the other four dimensions of EB or EVC (Table 1). Specifically, the average value of FF for only-children is higher than that of non-only-children. The average value of NE for non-only-children is higher than that of only-children.

**Table 1:** Differences in FF, exercise behavior, and exercise value cognition.

**Variable**	**Only and non-only-child (** ***M*** **±** ***SD*** **)**		***t***	***p***
	**Only-Child (*n* = 135)**	**Non-Only-Child (*n* = 367)**		
FF	37.11 ± 7.96	34.54 ± 8.37	3.080	0.002
EVC	21.99 ± 5.07	22.01 ± 4.57	−0.054	0.957
AE	38.50 ± 11.81	39.08 ± 10.19	−0.542	0.588
AC	22.42 ± 6.37	22.54 ± 5.64	−0.204	0.839
EP	12.15 ± 4.30	11.80 ± 3.78	0.891	0.374
SI	13.48 ± 3.61	13.44 ± 3.48	0.089	0.929
NE	11.69 ± 2.46	12.30 ± 3.06	−2.085	0.038
EB	98.24 ± 23.57	99.17 ± 20.19	−0.436	0.663

*FF, family function. EVC, exercise value cognition. AE, autonomous exercise. AC, attention control. EP, exercise planning. SI, situation induction. NE, negative exercise. EB, exercise behavior*.

### Correlation Analysis

The results of the Pearson correlation analysis (Table 2) showed that autonomous exercise, attention control, exercise planning, situation induction, and exercise behavior were each significantly positively correlated with FF and exercise value cognition (*p* < 0.01) in the full sample, only-child subsample, and non-only-child subsample. Since negative exercise behavior was not significantly correlated with FF or exercise value cognition, this variable was not included in subsequent analysis.

**Table 2:** Pearson correlation analysis of FF, exercise value cognition, and exercise behavior.

**Sample**	**Variable**	**AE**	**AC**	**EP**	**SI**	**NE**	**EB**
Only-Child	FF	0.319^**^	0.434^**^	0.295^**^	0.344^**^	0.061	0.390^**^
	EVC	0.527^**^	0.471^**^	0.474^**^	0.498^**^	0.096	0.564^**^
Non-Only-Child	FF	0.407^**^	0.426^**^	0.338^**^	0.418^**^	−0.024	0.456^**^
	EVC	0.319^**^	0.419^**^	0.189^**^	0.367^**^	0.077	0.388^**^
Full sample	FF	0.371^**^	0.421^**^	0.325^**^	0.394^**^	−0.02	0.426^**^
	EVC	0.381^**^	0.432^**^	0.273^**^	0.404^**^	0.078	0.440^**^

*N_only−child_ = 135, N_non−only−child_ = 367, N_fullsample_ = 504*,

** *p < 0.01. FF, family function; EVC, exercise value cognition; AE, autonomous exercise; AC, attention control; EP, exercise planning; SI, situation induction; NE, negative exercise; EB, exercise behavior*.

### Mediation Analysis of EVC

**Figure 1:**
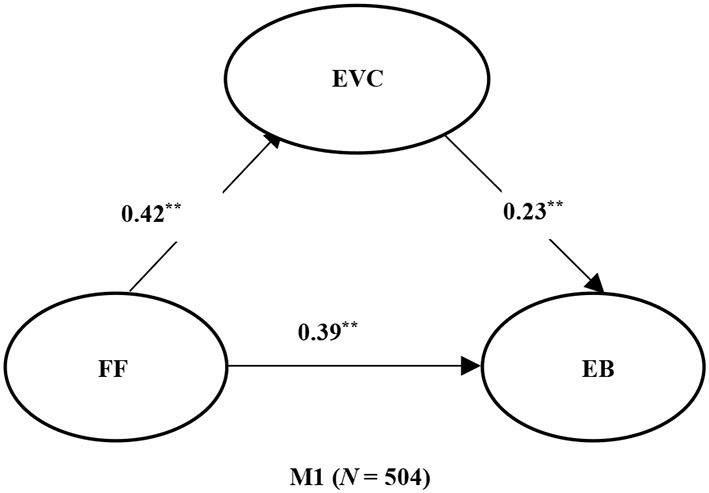
Path relationship diagram of Mediation model. **p < 0.01. FF, family function. EVC, exercise value cognition; EB, exercise behavior. The values in the figure are standardized path coefficients.

As reported in Table 3, the model fit indicator values fell within acceptable ranges, thus indicating relatively good model fit. The path analysis results show that (see Figure 1) FF is a significantly positive predictor of exercise behavior, both directly and through exercise value cognition.

**Table 3:** Fitting indicators of the mediation model of exercise value cognition.

**Model**	***x^**2**^/df***	**TLI**	**CFI**	**GFI**	**RMSEA**	**SRMR**
M1	4.242	0.930	0.942	0.901	0.080	0.069
Reference	<5.00	>0.90	>0.90	>0.90	<0.08	<0.05

As reported in Table 4, the bootstrapping results show that the CI of the indirect effect of path “FF → EVC → EB” does not contain 0, which implies that the mediating effect is significant in mediation model. Combining these results with the path analysis findings, exercise value cognition plays a partial mediating role in M1, with an effect size of 0.09.

**Table 4:** Bootstrap analysis of the significance test of the mediating effect.

**Model**	**Effects**	**Estimate**	**Product of Coefficient**		**Bootstrapping**	
					**Bias-Corrected 95% CI**	
			**SE**	***Z***	**Lower**	**Upper**
M1	Total effect	0.480	0.049	9.796	0.384	0.578
	Indirect effect (FF → EVC → EB)	0.094	0.023	4.087	0.053	0.143
	Direct effect (FF → EB)	0.386	0.054	7.148	0.284	0.498

*SE, standard error; CI, confidence interval. Total effect = Indirect effect + Direct effect*.

### Moderation Analysis of Only-Child Status

The fit indicators of the three models all reached acceptable levels (see Table 5). The results show that there is no significant difference between M2 and M3 (Δ*x*^2^ = 17.105, Δ*df* = 12, *p* > 0.05), but it reveals a significant difference between M2 and M4 (Δ*x*^2^ = 34.720, Δ*df* = 15, *p* < 0.01) and between M3 and M4 (Δ*x*^2^ = 17.615, Δ*df* = 3, *p* < 0.01). The results show that the models are invariant and the path coefficients between groups have significant differences.

**Table 5:** Fitting indicators of nested model comparison.

**Model**	***x^**2**^/df***	**TLI**	**CFI**	**GFI**	**RMSEA**	**SRMR**
M2	2.852	0.923	0.936	0.877	0.061	0.080
M3	2.760	0.927	0.935	0.873	0.059	0.078
M4	2.809	0.925	0.932	0.866	0.054	0.105
Reference	<5.00	>0.90	>0.90	>0.90	<0.08	<0.05

Taking M4 as the baseline model, the same method was used to test the equality of path coefficients for each research variable, so as to further test the difference between only-child and non-only-child on each path.

The results show significant differences between only-child and non-only-child in the regression coefficients of the two paths “FF → EB” and “EVC → EB” (Δ*x*^2^ = 7.789, Δ*df* = 1, *p* < 0.01; Δ*x*^2^ = 17.057, Δ*df* = 1, *p* < 0.01). However, there is no significant difference between only-child and non-only-child in the regression coefficient for path “FF → EVC” (Δ*x*^2^ = 0.378, Δ*df* = 1, *p* > 0.05).

**Figure 2:**
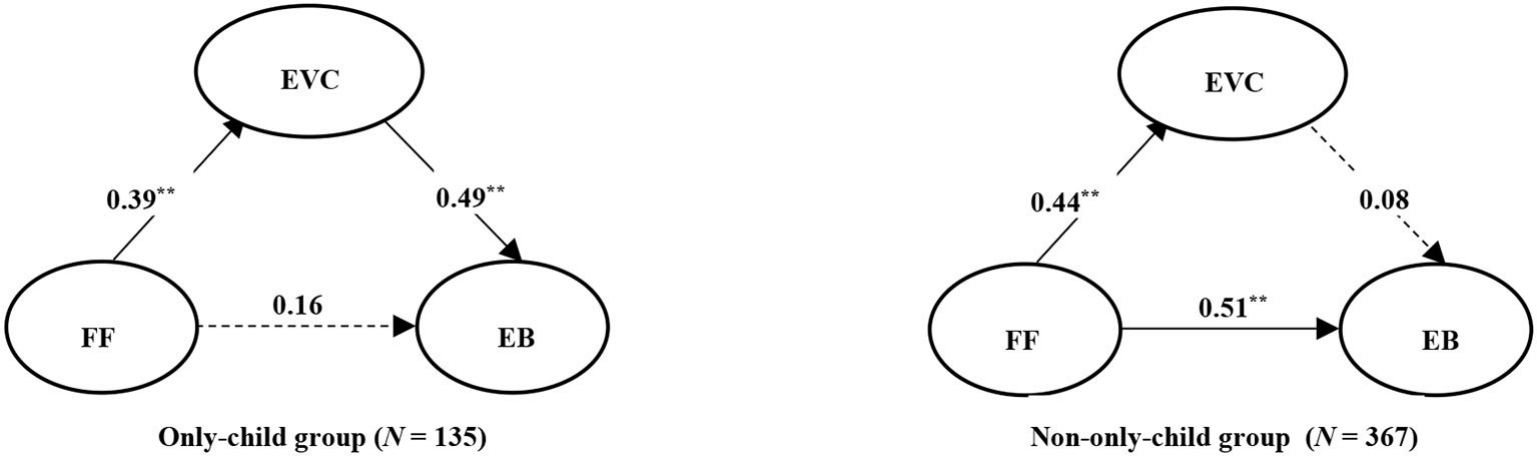
Path relationship diagram of Mediation model in two groups. **p < 0.001. FF, family function; EVC, exercise value cognition; EB, exercise behavior. The values in the figure are standardized path coefficients.

To further examine the mediating effect of exercise value cognition in only-child group and non-only-child group, bootstrapping was used to test the significance of the mediating effects (extract 2,000 bootstrap samples). The bootstrapping results show that the CI of the indirect effect in only-child group is [0.103, 0.333], and the CI of the indirect effect in non-only-child group is [-0.010, 0.083], meaning that the mediating effect is significant in only-child group. The path analysis results show that (see Figure 2) in only-child group, FF is a significantly positive predictor of exercise behavior only through exercise value cognition. In non-only-child group, FF is a significantly positive predictor of exercise value cognition and exercise behavior but not the latter through the former (i.e., the mediated relationship is not significant). Combining these results, exercise value cognition plays a complete mediating role in only-child group, with an effect size of 0.19.

## Discussion

### Differences Between Only-Child and Non-only-child in FF and Exercise Behavior

This study found that only-children and non-only-children differ significantly in FF and negative exercise, but not in exercise behavior overall, the four other dimensions of exercise behavior, and exercise value cognition. Relative to the non-only-child group, the only-child group has a higher average FF score and a lower average negative exercise score. These better results for only-child college students are consistent with prior findings. Against the background of the OCP and traditional cultures, the “pampered” one-child family model has become predominant in China. Under this model, the child not only receives more care from family members but also shoulders more expectations (Liu et al., 2011; Fang, 2018). Meanwhile, improvements to quality of life and the reform of educational and health concepts are spurring family members to pay more attention to cultivating the health habits and behaviors their children. These developments are reflected in only-child college students showing a higher level of FF and lower engagement in negative physical exercise behaviors, relative to their non-only-child peers. The results also provide support for the quantity–quality model of Becker and Lewis (1973). In addition, with the popularization of health education and the strengthening of exercise awareness in China, exercise behavior and exercise value cognition might be affected by demographic factors such as gender and grade (Cameron et al., 2013; Lemoyne et al., 2016); however, only-child and non-only-child students did not significantly differ in these variables.

Notably, a Chinese study had not found significant differences between only-children and non-only-children in FF (Sheng, 2015). There are two plausible reasons for the inconsistency between the results of this study and earlier findings. One is methodological differences, for instance in terms of sample selection, research tools, and research design. The other is that the FFs of only-children mostly depend on the intergenerational intimacy, whereas in non-only-child families, there might be a certain compensatory effect on communication between siblings. In view of the inconsistency of research results, there is a need for large-sample research to conduct in-depth investigation of FFs under different family structures in China.

### The Mediating Role of EVC and the Moderating Effect of Only and Non-only-child

First, the study found that FF is significantly positively correlated with exercise value cognition, exercise behavior overall, and four dimensions of exercise behavior (autonomous exercise, attention control, exercise planning, and situation induction). The significant predictive effects of FF on the exercise behavior and exercise value cognition of college students, respectively, support Hypothesis 1 and Hypothesis 2. Moreover, the significant predictive effect of exercise value cognition on exercise behavior supports Hypothesis 3. The mediating effect test found that exercise value cognition partially mediates the influence of FF on the exercise behavior of college students, meaning that this relationship is both direct and indirect (through exercise value cognition), thus supporting Hypothesis 4. The results of this study are consistent with the prior findings that the better an individual's cognition of FF, the more positive is their exercise value cognition, and the easier it is for them to plan and engage in regular physical exercise. For example, Berge et al. (2013) found that FF could predict weight-related health behaviors of adolescents, such as diet, sedentary behavior, and physical exercise. A cross-sectional study by Haines et al. (2016) revealed that higher the FF level, the more actively adolescents engage in exercise behavior. Ali and Malik (2015) found that FF and health promotion behaviors have a certain predictive effect on individual life quality. They pointed that FF indirectly impacts on individual life quality through health promotion behaviors. Meanwhile, the results of this study are consistent with family system theory and somewhat verify the process-orientation theory. FFs could affect the understanding and engagement of children in health-related behaviors. The results of this study suggest that the family environment positively influences the cultivation of individual exercise behaviors. Maintaining sound communication between family members and developing a healthy lifestyle can positively influence the exercise value cognition and exercise behavior of children.

Second, the study found that the mediating effect of exercise value cognition between FF and exercise behavior differs significantly between only-child and non-only-child college students. Specifically, exercise value cognition plays a complete mediating role in the only-child group but is non-significant in the non-only-child group. This finding supports Hypothesis 5 on the moderating role of only-child status. Further analysis revealed that only-child status regulated the two paths “FF → EB” and “EVC → EB.” This means that the intergroup differences mainly manifest in the influence of FF on exercise behavior and the influence of exercise value cognition on exercise behavior. These results show that exercise value cognition plays a greater mediating role between FF and exercise behavior in the only-child group. There are two main reasons for this. First, affected by the OCP, family education methods have undergone profound changes in China. Some concepts in family life and education have been weakened, such as the preference for a son and the prevailing importance of academic performance. The educational philosophy of Chinese parents has gradually shifted their main focus from intellectual development to comprehensive development. In China, compared with non-only-children, only-children will receive more care and love from their parents and even grandparents, which leads some only-children to cultivate specific behaviors and cognitive systems. For example, only-children differ significantly in terms of family-member interaction, family intimacy, and parental investment in education (Cameron et al., 2013; Hesketh et al., 2015). Based on these differences, it is easier for parents to notice the daily life of an only-child, such as their learning and exercise, enabling parents to respond promptly to specific situations. Second, Bandura (1986) posits that behavior, environment, and cognition are interdependent. Accordingly, exercise value cognition is likely to affect exercise behavior. Meanwhile, studies have found that, relative to a non-only-child, an only-child is typically able to form a better grasp of health-related knowledge, lifestyle, and skills in the course of growing up, making their exercise value cognition a greater predictor of exercise behavior (Hu and Yu, 2019).

In general, the moderating effect of only-child status provides useful guidance for the formulation of personalized physical exercise for college students. As only-children typically have a higher level of FF (including more positive emotional experiences, closer family relationships, lower error punishment rates, and a more democratic environment), focus should be placed on improving awareness of health among parents or family members, enhancing communication between families, and increasing family physical activities. Such efforts would help to provide better environmental support for the physical and mental health of children.

### Limitations and Implications

From the perspective of family system theory, this research reveals the mechanism through which FF influences on the exercise behavior of college students. However, the cross-sectional design adopted in this study and the results for the mediating effect cannot fully and concretely explain the relationships between the variables. Different family structures have different FFs. The one-child family includes multiple subsystems, such as “father–son/daughter” and “mother–son/daughter.” Therefore, future studies could explore the systematic changes in the exercise behaviors of college students over time, including how these are affected by various dimensions of family structure (e.g., only/non-only-child; divorced or widowed parents) and exercise value cognition. By clarifying the mechanism among these and related variables, empirical evidence can be generated and analyzed to inform the promotion of exercise behavior among college students. Scholars have already started to refine FFs based on family structure and the relationships between family members, and have begun developing valid and reliable tools for systematically evaluating the characteristics and mechanisms of individual FFs (Liu et al., 2011). In line with this trend, future studies could further investigate the influence of FFs on the exercise behaviors of college students under different family structures, and explore the mechanism of this relationship by using contextual measurement techniques. In addition, considering the representativeness of the samples in this study, future studies will expand the scope of the investigation, increase the representativeness of the samples, and examine the relationship between FF and exercise behavior.

## Conclusion

This study drew the following conclusions. First, the FF of college students is a significantly positive predictor of their exercise value cognition and exercise behavior, and also indirectly affects exercise behavior through exercise value cognition. Second, college the exercise value cognition of students plays a partial mediating role between FF and exercise behavior in the full sample and a completely mediating role in this relationship in the only-child subsample. Finally, only-child status significantly moderates the mediating effect of exercise value cognition between FF and exercise behavior, and exercise value cognition plays a greater mediating role between FF and exercise behavior in the only-child subsample.

## Data Availability Statement

The original contributions presented in the study are included in the article/Supplementary Material, further inquiries can be directed to the corresponding author/s.

## Ethics Statement

Ethical review and approval was not required for the study on human participants in accordance with the local legislation and institutional requirements. Written informed consent for participation was not required for this study in accordance with the national legislation and the institutional requirements.

## Author Contributions

MW conceived and designed the research, undertook data analysis, and wrote the Chinese manuscript. PY-W and MW wrote and supplemented the English manuscript. JY participated in the manuscript revision. MW and XL participated in the collection and sorting of data. All authors contributed to the article and approved the submitted version.

## Conflict of Interest

The authors declare that the research was conducted in the absence of any commercial or financial relationships that could be construed as a potential conflict of interest.

## Publisher's Note

All claims expressed in this article are solely those of the authors and do not necessarily represent those of their affiliated organizations, or those of the publisher, the editors and the reviewers. Any product that may be evaluated in this article, or claim that may be made by its manufacturer, is not guaranteed or endorsed by the publisher.

## References

[B1] Ali S. Malik J. A. (2015). Consistency of prediction across generation: explaining quality of life by family functioning and health-promoting behaviors. Qual. Life Res. 24, 2105–2112. 10.1007/s11136-015-0942-6 25724696

[B2] Bandura A. (1986). Social Foundations of Thought and Action: A Social Cognitive Theory. Upper Saddle River, NJ: Prentice Hall.

[B3] Beavers W. R. Hampson R. (2000). The beavers systems model of family functioning. J. Fam. Ther. 22, 128–143. 10.1111/1467-6427.00143

[B4] Becker G. S. Lewis H. G. (1973). On the interaction between the quantity and quality of children. J. Polit. Econ. 81:113.12178275

[B5] Berge J. M. Wall M. Larson N. Loth K. A. Neumark-Sztainer D. (2013). Family functioning: associations with weight status, eating behaviors, and physical activity in adolescents. J. Adolesc. Health 52, 351–357. 10.1016/j.jadohealth.2012.07.006 23299010 PMC3580029

[B6] Cadilhac D. A. Kilkenny M. F. Johnson R. Wilkinson B. Lalor E. (2015). The know your numbers (KYN) program 2008 to 2010: impact on knowledge and health promotion behavior among participants. Int. J. Stroke 10, 110–116. 10.1111/ijs.12018 23490310

[B7] Cameron L. Erkal N. Gangadharan L. Meng X. (2013). Little emperors: behavioral impacts of China's one-child policy. Science 339, 953–957. 10.1126/science.1230221 23306438

[B8] Chi X. Huang L. Wang J. Zhang P. (2020). The prevalence and socio-demographic correlates of depressive symptoms in early adolescents in china: differences in only child and non-only child groups. Int. J. Env. Res. Public Health 17:438. 10.3390/ijerph17020438 31936468 PMC7014354

[B9] DeVellis R. F. (2003). Scale Development theory and Applications, 2nd Edn. London:sage.

[B10] Dewar D. L. Plotnikoff R. C. Morgan P. J. Okely A. D. Costigan S. A. Lubans D. R. (2013). Testing social-cognitive theory to explain physical activity change in adolescent girls from low-income communities. Res. Q. Exerc. Sport 84, 483–491. 10.1080/02701367.2013.842454 24592778

[B11] Egawa K. (2013). Correlation between family function and spontaneous physical activity habit in parent and child: an exercise ecological study from a community random sample. Jpn. J. Hum. Growth Dev. Res. 2013, 10–17. 10.5332/hatsuhatsu.2013.10

[B12] Epstein N. B. Baldwin L. M. Bishop D. S. (1983). The McMaster family assessment device. J. Marital Fam. Ther. 9, 171–180. 10.1111/j.1752-0606.1983.tb01497.x

[B13] Essiet I. A. Baharom A. Shahar H. K. Uzochukwu B. (2017). Application of the socio-ecological model to predict physical activity behaviour among Nigerian University students. Pan. Afr. Med. J. 26:110. 10.11604/pamj.2017.26.110.10409 28533833 PMC5429408

[B14] Fang S. (2018). Only-Child Parents and their Only Children: How the One Child Policy Influences Chinese Family Functioning and Child Outcomes. (Doctoral dissertation), Fordham University. Retrieved from https://search.proquest.com/dissertations-theses/only-child-parents-their-children-how-one-policy/docview/2056877614/se-2?accountid=10659

[B15] Fang X. Xu J. Sun L. Zhang J. (2004). Family functioning: theory, influencing factores, and its relationship with adolescent social adjustment. Adv. Psychol. Sci. 12, 544–553. 10.3969/j.issn.1671-3710.2004.04.009 [in Chinese]

[B16] Ferguson K. J. Yesalis C. E. Pomrehn P. R. Kirkpatrick M. B. (1989). Attitudes, knowledge, and beliefs as predictors of exercise intent and behavior in school children. J. Sch. Health 59, 112–115. 10.1111/j.1746-1561.1989.tb04675.x 2704183

[B17] Haines J. Rifas-Shiman S. L. Horton N. J. Kleinman K. Bauer K. W. Davison K.. (2016). Family functioning and quality of parent-adolescent relationship: cross-sectional associations with adolescent weight-related behaviors and weight status. Int. J. Behav. Nutr. Phys. 13:68. 10.1186/s12966-016-0393-7 27301414 PMC4908682

[B18] Hesketh T. Zhou X. Wang Y. Zhou X. Wang Y. (2015). The end of the one-child policy: lasting implications for China. J. Am. Med. Assoc. 314, 2619–2620. 10.1001/jama.2015.16279 26545258

[B19] Hu P. Yu F. (2019). A study about the restrictive factors on physical exercise of the middle school students—an HLM model based on CEPS (2014-2015). China Sport Sci. 39, 76–84. 10.16469/j.css.201901010

[B20] Jackson D. L. (2003). Revisiting sample size and number of parameter estimates: some support for the N:q hypothesis. Struct. Equ. Model. 10, 128–141. 10.1207/S15328007SEM1001_6

[B21] Kilanowski J. F. (2017). Breadth of the socio-ecological model. J Agromed. 22, 295–297. 10.1080/1059924X.2017.1358971 28742433

[B22] Kim S. Ahn T. Fouad N. (2015). Family influence on Korean students' career decisions. J. Career Assess. 24, 513–526. 10.1177/1069072715599403

[B23] Knol L. L. Myers H. H. Black S. Robinson D. Awololo Y. Clark D.. (2016). Development and feasibility of a childhood obesity prevention program for rural families: application of the social cognitive theory. Am. J. Health Educ. 47, 204–214. 10.1080/19325037.2016.1179607 28392882 PMC5383209

[B24] Lemoyne J. Valois P. Wittman W. (2016). Analyzing exercise behaviors during the college years: results from latent growth curve analysis. PLoS ONE 11:e0154377. 10.1371/journal.pone.0154377 27124179 PMC4849640

[B25] Li X. Zhang Y. Feng C. Gao Q. Xie G. (2018). A study on the status and influential factors of health literacy among junior college students in a medical college in Shijiazhuang City. Chin. J. Health Educ. 34, 703–708. 10.16168/j.cnki.issn.1002-9982.2018.08.007

[B26] Liu H. (2019). Parenting practices and the class differentiation in China. Soc. Sci. 8, 62–75. 10.13262/j.bjsshkxy.bjshkx.190806

[B27] Liu X. Zhang J. Yeh C. Y. L. (2011). Investigating the validity of a multirater assessment of family functioning in China. Soc. Behav. Pers. 39, 773–783. 10.2224/sbp.2011.39.6.773

[B28] Loprinzi P. D. (2015). Association of family functioning on youth physical activity and sedentary behavior. J. Phys. Act. Health 12, 642–648. 10.1123/jpah.2014-0031 25110353

[B29] MacKinnon D. P. Lockwood C. M. Hoffman J. M. West S. G. Sheets V. (2002). A comparison of methods to test mediation and other intervening variable effects. Psychol. Methods 7, 83–104. 10.1590/S1516-635X2013000400006 11928892 PMC2819363

[B30] MacKinnon D. P. Lockwood C. M. Williams J. (2004). Confidence limits for the indirect effect: distribution of the product and resampling methods. Multivar. Behav. Res. 39, 99–128. 10.1207/s15327906mbr3901_4 20157642 PMC2821115

[B31] Mao R. (2003). The establishment and test of a nine-factor model of “exercise attitude-behavior” among adolescents (Master's thesis). Beijing Sport University, Beijing, China.

[B32] Martín-Matillas M. Ortega F. B. Chillon P. Pérez Isaac J. Ruiz J. R. Castillo R.. (2011). Physical activity among Spanish adolescents: relationship with their relatives' physical activity—the AVENA Study. J. Sports Sci. 29, 329–336. 10.1080/02640414.2010.523091 21184343

[B33] Maruf F. A. Chianakwana C. Hanif S. (2017). Perception, knowledge, and attitude toward physical activity behavior: implications for participation among pregnant women. J Womens Health Phys. Ther. 41, 1–8. 10.1007/s40292-017-0235-y 29082466

[B34] Miller I. W. Epstein N. B. Bishop D. S. Keitner G. I. (1985). The McMaster family assessment device: reliability and validity. J Marital Fam. Ther. 11, 345–356. 10.1111/j.1752-0606.1985.tb00028.x25385473

[B35] Miller J. M. Wolfson J. Laska M. N. Nelson T. F. Pereira M. A. Neumark-Sztainer D. (2019). Factor analysis test of an ecological model of physical activity correlates. Am. J. Health Behav. 43, 57–75. 10.5993/AJHB.43.1.6 30522567 PMC6296241

[B36] Rausch J. C. Berger-Jenkins E. Nieto A. R. McCord M. Meyer D. (2015). Effect of a school-based intervention on parents' nutrition and exercise knowledge, attitudes, and behaviors. Am. J. Health Educ. 46, 33–39. 10.1080/19325037.2014.977411

[B37] Schmidt L. Rempel G. Murray T. C. McHugh T. L. Vallance J. K. (2016). Exploring beliefs around physical activity among older adults in rural Canada. Int. J. Qual. Stud. Health Well Being 11:32914. 10.3402/qhw.v11.32914 27834180 PMC5105319

[B38] Schreiber J. B. Nora A. Stage F. K. Barlow E. A. King J. (2006). Reporting structural equation modeling and confirmatory factor analysis results: a review. J. Educ. Res. 99, 323–338. 10.3200/JOER.99.6.323-338

[B39] Schumacker R. E. Lomax R. G. (2016). A Beginner's Guide to Structural Equation Modeling, 4th Edn. New York, NY: London: Routledge.

[B40] Schwab J. J. Gray-Ice H. M. (2000). Family Functioning-The General Living Systems Research Model. New York, NY: Kluwer Academic/Plenum Publishers. 10.1176/appi.ps.52.6.849

[B41] Shek D. T. (2002). Family functioning and psychological well-being, school adjustment, and problem behavior in Chinese adolescents with and without economic disadvantage. J. Genet. Psychol. 163, 497–500. 10.1080/00221320209598698 12495233

[B42] Sheng Y. (2015). Research on the relationship between physical exercise motivation and physical exercise behavior of college students in Yangzhou University (Master's thesis). Yangzhou University, Yangzhou, China.

[B43] Sicilia A. Águila C. Cornelio; Posse M. Alcaraz-Ibáñez M. (2020). Parents' and peers' autonomy support and exercise intention for adolescents: integrating social factors from the self-determination theory and the theory of planned behaviour. Int. J. Environ. Res. Public Health. 17:5365. 10.3390/ijerph17155365 32722479 PMC7432024

[B44] Skinner H. Steinhauer P. (2000). Family assessment measure and process model of family functioning. J. Fam. Ther. 22, 190–210. 10.1111/1467-6427.00146

[B45] Xiong M. Liu H. (2013). Effects of exercise knowledge popularity through public speeches on exercise motivation of undergraduate students. J. Wuhan Inst. Phys Educ. 47, 75–79. 10.15930/j.cnki.wtxb.2013.07.014

[B46] Yaffe Y. (2018). Physical activity among Israeli-Arab adolescent males: how do parenting styles matter? Am. J. Mens. Health 12, 2037–2043. 10.1177/1557988318790881 30043663 PMC6199442

[B47] Yao J. (2013). Research on the relationship between exercise value cognition and exercise behavior of community fitness crowd in Shanghai (Master's thesis). Shanghai Normal University, Shanghai, China.

[B48] Zhao L. Zhou M. (2018). Do only children have poor vision? Evidence from China's one-child policy. Health Econ. 27, 1131–1146. 10.1002/hec.3661 29682832

[B49] Zhu Q. Sun J. (2016). Influences of modern family functions on family keep-fit activities. J. Shenyang Sport Univ. 35, 42–46. 10.3969/j.issn.1004-0560.2016.03.008

