# Supplementary material for: The Family Function and Exercise Behavior of Chinese College Students: A Moderated Mediation Model of Exercise Value Cognition and Only-Child Status
Source: Front Psychol. 2021 Aug 27;12:644742. doi: 10.3389/fpsyg.2021.644742 (PMC8432938; doi:10.3389/fpsyg.2021.644742)
Supplement: Supplementary file 1 [file Table_1.DOCX]

**1 Family Function Scale**

1.When we encounter problems or difficulties, my family and I will solve these problems together.

2.We are sure that the family members have fulfilled their family responsibilities.

3.We can do anything in my family.

4.The relationship between family members is very close.

5. Family members can share interests and hobbies with each other.

6.Family members like to spend their spare time together.

7.In the family, the friends of each family member will be warmly received.

**2 Exercise Value Cognition Scale**

1. I think physical exercise is a good fitness and recreational activity.

2. I think physical exercise is good for physical and mental health.

3. Physical exercise enriches my life.

4. Physical exercise is one of my hobbies.

**3 Exercise Behavior Scale**

**(1) Autonomous Exercise**

1.Whether the sports skills of a class are well mastered, I will review it after class.

2.When the effect of physical education is not good, I will reflect on it.

3.In the physical education class, I will actively strive for opportunities to show myself.

4.Whenever I master a sports skill, I will discuss it with my teacher or classmates.

5.I often invite my partners to do extracurricular physical exercises.

6.I often challenge the limit of my strength during the exercise.

7.I can prevent the injury from happening again by summing up experience after the sports injury.

8.If I see my friends going to exercise, I will want to exercise.

9.I like to watch games or check sports related information.

**(2) Attention control**

1.I am very focused in physical education class.

2.In the process of physical education, I can overcome interference.

3.When learning sports skills, I can always devote myself to it.

4.No matter how many difficulties I may face, I will keep exercising.

5.My exercise goals will always be achieved through hard work.

**(3) Plan Exercise**

1.I always make exercise plans.

2.I exercise every day.

3.I often participate in sports competitions.

**(4) Situation induction**

1.I will exercise before the physical education exam (or sports competition).

2.If I do not exercise for a long time, I will remind myself to participate in physical exercise.

3.For some reasons (such as illness, obesity, etc.), I was forced to keep exercising.

**(5) Negative exercise**

1.When I meet with difficulties in physical education, I am ashamed to ask for help.

2.I often fail to complete the exercise program.

3.I am ashamed to ask the teachers for help, even when I meet with difficulties in skills learning.

**4 EFA of Exercise Behavior Scale**

| **Table S1. KMO and Bartlett's Test** | | |
| --- | --- | --- |
| Kaiser-Meyer-Olkin Measure of Sampling Adequacy. | | .922 |
| Bartlett's Test of Sphericity | Approx. Chi-Square | 2419.893 |
|  | df | 253 |
|  | Sig. | .000 |

| **Table S2. Total Variance Explained** | | | | | | | | | |
| --- | --- | --- | --- | --- | --- | --- | --- | --- | --- |
| Component | Initial Eigenvalues | | | Extraction Sums of Squared Loadings | | | Rotation Sums of Squared Loadings | | |
|  | Total | % of Variance | Cumulative % | Total | % of Variance | Cumulative % | Total | % of Variance | Cumulative % |
| 1 | 8.964 | 38.975 | 38.975 | 8.964 | 38.975 | 38.975 | 4.930 | 21.437 | 21.437 |
| 2 | 1.732 | 7.531 | 46.507 | 1.732 | 7.531 | 46.507 | 3.602 | 15.660 | 37.097 |
| 3 | 1.311 | 5.700 | 52.207 | 1.311 | 5.700 | 52.207 | 2.501 | 10.874 | 47.971 |
| 4 | 1.251 | 5.437 | 57.644 | 1.251 | 5.437 | 57.644 | 1.835 | 7.977 | 55.947 |
| 5 | 1.098 | 4.773 | 62.417 | 1.098 | 4.773 | 62.417 | 1.488 | 6.470 | 62.417 |
| 6 | .896 | 3.894 | 66.312 |  |  |  |  |  |  |
| 7 | .835 | 3.629 | 69.941 |  |  |  |  |  |  |
| 8 | .719 | 3.126 | 73.067 |  |  |  |  |  |  |
| 9 | .689 | 2.994 | 76.060 |  |  |  |  |  |  |
| 10 | .654 | 2.843 | 78.903 |  |  |  |  |  |  |
| 11 | .613 | 2.666 | 81.569 |  |  |  |  |  |  |
| 12 | .569 | 2.473 | 84.042 |  |  |  |  |  |  |
| 13 | .485 | 2.110 | 86.152 |  |  |  |  |  |  |
| 14 | .472 | 2.050 | 88.202 |  |  |  |  |  |  |
| 15 | .425 | 1.847 | 90.049 |  |  |  |  |  |  |
| 16 | .407 | 1.769 | 91.819 |  |  |  |  |  |  |
| 17 | .359 | 1.559 | 93.378 |  |  |  |  |  |  |
| 18 | .318 | 1.382 | 94.760 |  |  |  |  |  |  |
| 19 | .299 | 1.302 | 96.061 |  |  |  |  |  |  |
| 20 | .259 | 1.128 | 97.189 |  |  |  |  |  |  |
| 21 | .240 | 1.045 | 98.234 |  |  |  |  |  |  |
| 22 | .218 | .949 | 99.183 |  |  |  |  |  |  |
| 23 | .188 | .817 | 100.000 |  |  |  |  |  |  |
| Extraction Method: Principal Component Analysis. | | | | | | | | | |

| **Table S3. Rotated Component Martix^a^** | | | | | |
| --- | --- | --- | --- | --- | --- |
|  | component | | | | |
|  | 1 | 2 | 3 | 4 | 5 |
| item-12 | .764 |  |  |  |  |
| item-10 | .735 |  |  |  |  |
| item-18 | .715 |  |  |  |  |
| item-28 | .695 |  |  |  |  |
| item-26 | .645 |  |  |  |  |
| item-29 | .635 |  |  |  |  |
| item-1 | .634 |  |  |  |  |
| item-25 | .611 |  |  |  |  |
| item-16 | .565 |  |  |  |  |
| item-2 |  | .844 |  |  |  |
| item-3 |  | .815 |  |  |  |
| item-4 |  | .798 |  |  |  |
| item-5 |  | .637 |  |  |  |
| item-8 |  | .502 |  |  |  |
| item-11 |  |  | .810 |  |  |
| item-9 |  |  | .657 |  |  |
| item-21 |  |  | .590 |  |  |
| item-20 |  |  |  | .741 |  |
| item-17 |  |  |  | .639 |  |
| item-19 |  |  |  | .555 |  |
| item-7 |  |  |  |  | .735 |
| item-6 |  |  |  |  | .602 |
| item-23 |  |  |  |  | .582 |
| Extraction Method: Principal Component Analysis.  Rotation Method: Varimax with Kaiser Normalization. | | | | | |
| Rotation converged in 8 iterations. | | | | | |

**5 The gender difference of the variables and tested the moderating effect by multi-group comparison**

Having identified the unbalanced gender composition of the sample during data analysis, we sought to ensure the study’s scientific rigor by testing for gender difference in the variables and testing the moderating effect by multi-group comparison. The results of the independent sample T test (Table S4) showed that male and female college students differed significantly in autonomous exercise (*p* = 0.001 < 0.01), attention control (*p* = 0.017 < 0.05), exercise planning (*p* = 0.001 < 0.01), situation induction (*p* = 0.040 < 0.05), and exercise behavior (*p* = 0.001 < 0.01). The difference in exercise value cognition between male and female college students was only marginally significant (*p* = 0.049 < 0.05).

**TABLE S4.** Differences in family function, exercise behavior and exercise value cognition.

| **Variable** | **gender (*M±SD*)** | | ***t*** | ***p*** |
| --- | --- | --- | --- | --- |
|  | **male (*n* = 165)** | **female (*n* = 339)** |  |  |
| FF | 35.42±9.38 | 35.11±7.78 | 0.391 | 0.696 |
| EVC | 22.57±4.78 | 21.69±4.67 | 1.974 | 0.049 |
| AE | 41.06±10.05 | 37.83±10.79 | 3.226 | 0.001 |
| AC | 23.37±5.91 | 22.05±5.76 | 2.391 | 0.017 |
| EP | 12.68±3.77 | 11.48±3.95 | 3.242 | 0.001 |
| SI | 13.90±3.21 | 13.22±3.63 | 2.064 | 0.040 |
| NE | 12.46±2.49 | 11.97±3.10 | 1.768 | 0.078 |
| EB | 103.48±19.75 | 96.56±21.47 | 3.486 | 0.001 |

The multi-group analysis technique was adopted to test the moderating effect of gender using nested models. We first constructed an unconstrained model that freely estimates each path coefficient (M_1). We then built model M_2, with equal measurement weights of the two groups, based on M_1. Finally, we built model M_3, with the structural weights of the two groups also equal, based on M_2. The fit indicator values were acceptable for all three models (Table S5). The chi-square difference value (△*x*^2^) was adopted for differential analysis of the nested models. The results revealed significant differences between M_1 and M_2 (△*x*^2^ = 23.996, △*df* = 12, *p* = 0.020), between M_1 and M_3 (△*x*^2^ = 30.475, △*df* = 15, *p* = 0.010), and between M_2 and M_3 (△*x*^2^ = 6.479, △*df* = 3, *p* = 0.090). Therefore, the models are not invariant and the path coefficients between groups are not significantly different.

In summary, gender does not have a moderating role in our theoretical model, consistent with the view of previous studies. Therefore, we decided not to include this analysis of gender in the manuscript.

**Table S5.** Fitting indicators of multi-group test models.

| Model | ***x^2^/df*** | **NFI** | **CFI** | **GFI** | **RMSEA** | **SRMR** |
| --- | --- | --- | --- | --- | --- | --- |
| M_1 | 2.839 | 0.908 | 0.938 | 0.877 | 0.061 | 0.073 |
| M_2 | 2.785 | 0.903 | 0.936 | 0.871 | 0.060 | 0.081 |
| M_3 | 2.775 | 0.902 | 0.935 | 0.869 | 0.059 | 0.094 |
| Reference | ＜5.00 | ＞0.90 | ＞0.90 | ＞0.90 | ＜0.08 | ＜0.05 |

**6 The specific instructions of M2, M3 and M4 in the manuscript**

**(1) The model of two groups**


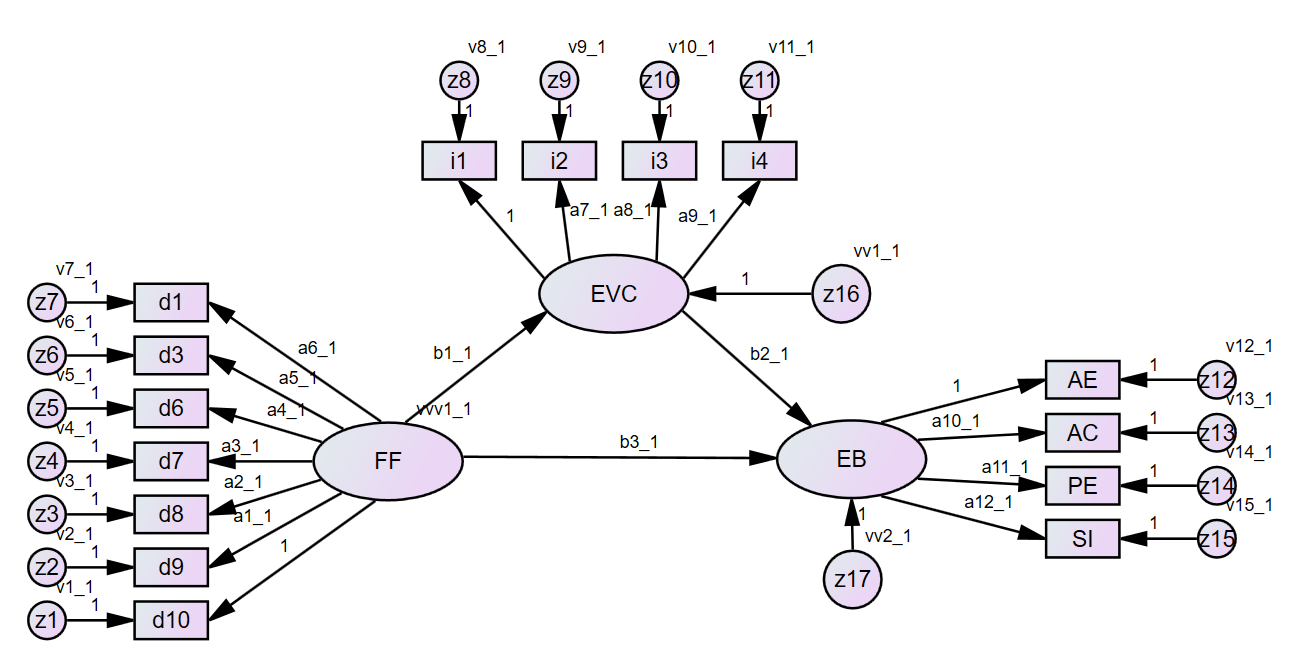


**Figure S1**. Only child group model.


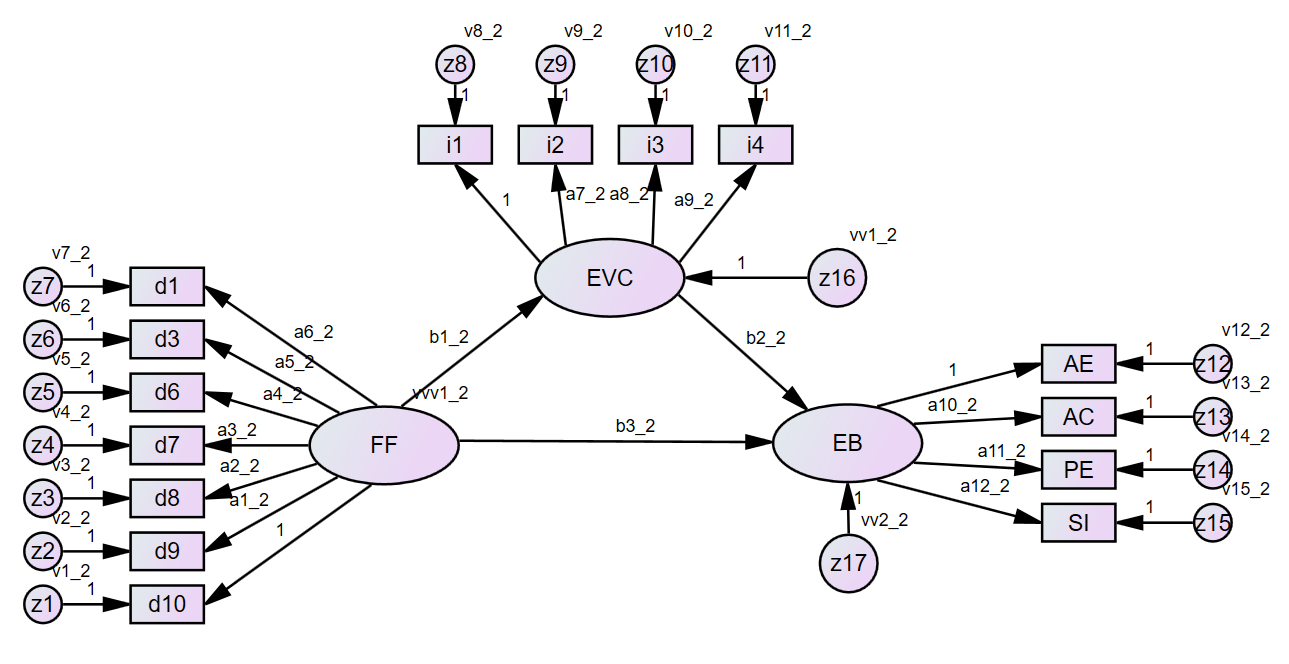


**Figure S2.** Non-only child group model.

**Table S6.** Related parameter description

| parameter name | Only child group | Non-only child group |
| --- | --- | --- |
| measurement weights | a1_1  a2_1  a3_1  a4_1  a5_1  a6_1  a7_1  a8_1  a9_1  a10_1  a11_1  a12_1 | a1_2  a2_2  a3_2  a4_2  a5_2  a6_2  a7_2  a8_2  a9_2  a10_2  a11_2  a12_2 |
| structural weights | b1_1  b2_1  b3_1 | b1_2  b2_2  b3_2 |

**(2) Nested model comparison settings**

**Table S7.** Nested model comparison settings

|  | M2（no parameter equality restriction between groups.） | M3（the measurement weights of the two groups are equal） | M4（the measurement weights of the two groups are equal, and structural weights of the two groups are equal） |
| --- | --- | --- | --- |
| measurement weights | No restriction on parameter equality between groups | a1_1 = a1_2  a2_1 = a2_2  a3_1 = a3_2  a4_1 = a4_2  a5_1 = a5_2  a6_1 = a6_2  a7_1 = a7_2  a8_1 = a8_2  a9_1 = a9_2  a10_1 = a10_2  a11_1 = a11_2  a12_1 = a12_2 | a1_1 = a1_2  a2_1 = a2_2  a3_1 = a3_2  a4_1 = a4_2  a5_1 = a5_2  a6_1 = a6_2  a7_1 = a7_2  a8_1 = a8_2  a9_1 = a9_2  a10_1 = a10_2  a11_1 = a11_2  a12_1 = a12_2 |
| structural weights | No restriction on parameter equality between groups | No restriction on parameter equality between groups | b1_1 = b1_2  b2_1 = b2_2  b3_1 = b3_2 |

**(3) Nested model comparison analysis steps**

**STEP 1** measurement invariance test of two groups

Null hypothesis: M2 = M3

Amos provides the chi-square difference (△*x^2^*) when comparing two nested models. If the significance of △*x^2^* < 0.05, the null hypothesis is rejected; if the significance of △*x^2^* > 0.05, the null hypothesis is accepted, which means that the models are cross-group invariant and the measurement weights of the two groups are not significantly different.

**STEP 2** Compare M2 and M4

Null hypothesis: M2 = M4

If the significance of △*x^2^* < 0.05, the null hypothesis is rejected, which means that the structural weights of the two groups may different; if the significance of △*x^2^* > 0.05, the null hypothesis is accepted.

**STEP 3** Compare M3 and M4

Null hypothesis: M3 = M4

If the significance of △*x^2^* < 0.05, the null hypothesis is rejected, which means that the structural weights of the two groups significantly different; if the significance of △*x^2^* > 0.05, the null hypothesis is accepted.

**STEP 4** Compare every structural weight of the two groups

Taking M2 as the baseline model, the same method was used to test the equality of path coefficients for each research variable, so as to further test the difference between only child and non-only child for each path.

**Table S8.** Nested model comparison settings

|  | M2 | M5 | M6 | M7 |
| --- | --- | --- | --- | --- |
| structural weights | No restriction on parameter equality between groups | b1_1 = b1_2 | b2_1 = b2_2 | b3_1 = b3_2 |

① Null hypothesis: M2 = M5

If the significance of △*x^2^* < 0.05, the null hypothesis is rejected, which means the structural weights of “FF→EVC” is significantly different; if the significance of △*x^2^* > 0.05, the null hypothesis is accepted.

②Null hypothesis: M2 = M6

If the significance of △*x^2^* < 0.05, the null hypothesis is rejected, which means the structural weights of “EVC→EB” is significantly different; if the significance of △*x^2^* > 0.05, the null hypothesis is accepted.

③Null hypothesis: M2 = M7

If the significance of △*x^2^* < 0.05, the null hypothesis is rejected, which means the structural weights of “FF→EB” is significantly different; if the significance of △*x^2^* > 0.05, the null hypothesis is accepted.

**6 The measures to protect the privacy of the subjects**

This study adopted the following measures to protect the privacy of the subjects:

1. Collect relevant information of subjects anonymously. Researchers and research team members cannot directly or indirectly identify subjects' names, ID numbers and IP information.

2. After the data is recovered, it will be kept by the project leader (corresponding author of this study).

3. The computer that saves the data is protected by a password. The folder where the data is stored will also be password protected. Members participating in the project can access the data after declaring the reason for use and submitting an application in writing.

4. The relevant data of this study will be kept for 3 years after the completion of the project. Then, the project leader will supervise the data user to delete the data and not back it up. After all the research is completed, project leader will delete the data in a unified way.
